# Curcumin liposomes interfere with quorum sensing system of *Aeromonas sobria* and *in silico* analysis

**DOI:** 10.1038/s41598-017-08986-9

**Published:** 2017-08-17

**Authors:** Ting Ding, Tingting Li, Zhi Wang, Jianrong Li

**Affiliations:** 1 School of Food Science and Technology, Jiangnan University, Wuxi, 214122 China; 2 Key Laboratory of Biotechnology and Bioresources Utilization (Dalian Minzu University), Ministry of Education, Dalian, Liaoning 116600 China; 3 College of Food Science and Technology, Bohai University, Food Safety Key Lab of Liaoning Province, National & Local Joint Engineering Research Center of Storage, Processing and Safety Control Technology for Fresh Agricultural and Aquatic Products, Jinzhou, Liaoning 121013 China; 4 School of Chemical and Environmental Engineering, Shanghai Institute of Technology, Shanghai, 201418 China

## Abstract

*Aeromonas sobria* is opportunistic pathogen frequently found in environment and food. Interfering with its quorum sensing (QS) system could be a promising way to alleviate its virulence. In this study, curcumin liposomes were prepared and their characteristics like particle size, zeta potential, PDI (Polymey Disperse Index), encapsulation efficiency and loading capacity were measured. The quorum sensing inhibitory effect of curcumin liposomes under sub-MIC (Minimum Inhibitory Concentration) on siderophore production, swimming and swarming motility, extracellular proteases, biofilm formation and AHLs (N-acylhomoserine lactones) production of *A. sobria* were also determined. The results showed that, the curcumin liposomes with high encapsulation capacity (84.51 ± 0.58%) were stable and homogeneous. QS-regulated phenotypes of the pathogen were significantly inhibited by curcumin liposomes. The *in silico* analysis revealed that the QS system of *A. sobria* may be inhibited by released curcumin from curcumin liposomes through interacting with the built LuxI type protein and blocking the production of AHLs.

## Introduction

*Aeromonas sobria* is a Gram-negative, rod-shaped, facultative anaerobic, motile, and flagellated bacterium of the Aeromonadaceae family^1^ *. A. sobria* usually thrives in fresh water, sea water, sewage, soil and food. As an opportunistic pathogen for animals and humans, it can cause extra-intestinal infections and gastrointestinal diseases^2^. It is also a causative bacterium of child diarrhea and soft-tissue infections in fish^3^. Currently, antibiotics and synthetic antibacterials are widely used to treat bacterial-caused diseases. However, improper use of antibiotics has created the problems of antibiotics resistance and environmental contamination.

Quorum sensing is a cell-to-cell communication mechanism, allowing bacteria to monitor their population density by secreting signaling molecules or autoinducers^4^. These chemical signals release from bacteria and accumulate in the environment, and once the threshold concentration reaches, they can bind to receptor proteins and trigger the expression of a series of QS targeting genes^5^. Most Gram-negative bacteria use N-acylhomoserine lactones (AHLs) as the major QS signal molecules, while Gram-positive bacteria use signal peptides to regulate physiological functions, such as bioluminescence, biosynthesis of antibiotic, release of virulence factors, biofilm formation, siderophore, extracellaluar proteases, swimming and swarming motility, *etc*.^6, 7^. Therefore, blocking the bacterial QS system has been considered as an alternative of traditional antibiotics, for it would not induce the resistance of bacteria due to its distinctive antibacterial mechanism^8^. It has been reported that *A. sobria* produces AHLs such as C4-HSL and C6-HSL to regulate its virulence factors^9^. Meanwhile, many natural products and synthetic materials such as iberin^10^, baicalein^11^, quercetin^12^, and 6-Gingerol^13^ have been proven to be quorum sensing inhibitors (QSIs). Among them, the brominated furanones such as furanone C-30 have been reported to inhibit QS-regulated biofilm formation and production of virulence factors^14^. However, their toxicity to mammalian cells has not been reported^15^. On the other hand, curcumin, the major ingredient from *Curcuma longa* has shown multiple pharmaceutical properties, such as antitumor^16, 17^, anti-HIV-1^18^, antibacterial^19^, *etc*. Curcumin has also been shown to have inhibitory effect on the virulence, QS, and biofilm initiation of *Pseudomonas aeruginosa* PAO1 in the whole plant and animal models^20^. Similarly, curcumin also exhibited inhibitory effect on the QS system of uropathogens, including *Escherichia coli*, *Proteus mirabilis*, *Pseudomonas aeruginosa* PAO1 and *Serratia marcescens* ^21^. More importantly, curcumin has been applied as a dietary supplement and alternative medicine, and it is believed to be safe and efficacy when administered at very high doses clinically^22^. However, the bioavailability of curcumin was limited due to its poor water solubility and fast metabolism^21, 23^. Liposomes have been effective carriers for hydrophobic drugs^24^. Additionally, the inhibitory effect of curcumin liposomes acted as QSIs on the QS system of *A. sobria* has not been demonstrated. Herein, in the present study, the curcumin liposomes were prepared to study the inhibitory effect on quorum sensing system of *A*. *sobria*. The possible inhibitory mechanism was further analyzed by molecular docking techniques.

## Materials and Methods

### Materials and bacterial strains

Curcumin, cholesterol, lecithin, 2, 3-bis (2-methoxy-4-nitro-5-sulfo-phenyl)-2H-tetrazolium-5- carboxanilide (XTT) and menadione were purchased from Sigma-Aldrich (St. Louis, MO, USA). Standard AHLs including N-butanoyl-L-homoserine lactone (C4-HSL), N-hexanoyl-L-homoserine lactone (C6-HSL), N-octanoyl-L-homoserine lactone (C8-HSL), N-decanoyl-L- homoserine lactone (C10-HSL), N-dodecanoyl-L-homoserine lactone (C12-HSL) and N-tetradecanoyl-L-homoserine lactone (C14-HSL) were also purchased from Sigma-Aldrich. The AHLs C4-HSL, C6-HSL, C8-HSL, C10-HSL, C12-HSL and C14-HSL were dissolved by methanol (at 20 µM). The strains *Chromobacterium violaceum* CV026 and *Agrobacterium tumefaciens* A136 were kind gifts from Dr. Yang (Xinjiang Shihezi University, Xinjiang, China). *C. violaceum* CV026 is a mini-Tn5 mutant deficient in AHLs production, but it responds to exogenous AHLs and produces violacein in purple color^25^. Herein, it was used as an indicator bacterium for short chain AHLs detection. *A. tumefaciens* A136, which carries a *lac*Z fusion to *tra*I, was used to detect long chain AHLs. It produces blue color in the presence of long chain AHLs and X-Gal (5-bromo-4- chloro-3-indoyl-β-D-galactopyranoside). *C. violaceum* CV026 was grown in 10 ml of Luria-Bertani (LB) (Beijing aoboxing bio-tech CO., LTD, China) medium containing 10 μl of kanamycin (20 μg/ml). *A. tumefaciens* A136 was grown in 10 ml of LB medium containing 10 μl of tetracycline (20 μg/ml) and 50 μl of spectinomycin (20 μg/ml). *A. sobria* was isolated and identified by our laboratory from deteriorated aquatic products. Curcumin was dissolved in 1% dimethyl sulfoxide (DMSO, dissolved in distilled water). Methanol was chromatographically pure and other chemicals were analytical reagents and all chemical reagents are commercially available.

### Preparation of curcumin liposomes

The curcumin liposomes were prepared by using a conventional thin film-dispersed method with slight modification^26^. A total of 100 mg lecithin, 42 mg cholesterol and 5 mg curcumin were dissolved into 10 ml chloroform, respectively. And 1 ml of each solution was added to a rotary flask and mixed for 5 min (30 °C, 140 bar, 90 r/min) to obtain a thin film. A total of 1 ml 5% glucose (w/v) was added to the film and mixed by rotary evaporation under normal pressure (50 °C) for 2 hours. Then the solution was sonicated using an ultrasonic cell disruptor (DS-650Y, Doosy, Shanghai, China) for 100 cycles (2 s on, 2 s off, 400 W) until the solution was homogeneous. The temperature was controlled by an ice bath. The curcumin liposomes were obtained by filtration through a micro-pore filter (0.22 µm).

### Determination of characteristics of curcumin liposomes

The transparent yellow solution of curcumin liposomes was obtained by sephadex gel column chromatography. The sample (0.1 ml) was added to 1.9 ml methanol for demulsification and the characteristics of curcumin liposomes were measured.

#### Measurement of encapsulation efficiency and loading capacity

The amount of entrapped curcumin was measured by high performance liquid chromatography (HPLC) at the detection wavelength of 425 nm, which was the maximum absorbance wavelength of curcumin. And it was analyzed quantitatively by comparing with a standard curve. The experiments were repeated three times. The loading capacity (LC %) and encapsulation efficiency (EE %) was derived by the equations below (1 and 2), respectively.

1 $${\rm{LC}} \% =({\rm{Total}}\,{\rm{amount}}\,{\rm{of}}\,{\rm{determined}}\,{\rm{curcumin}}/{\rm{Total}}\,{\rm{amount}}\,{\rm{of}}\,{\rm{dried}}\,{\rm{liposomes}})\times 100 \% $$

2 $${\rm{EE}} \% =({\rm{Total}}\,{\rm{amount}}\,{\rm{of}}\,{\rm{determined}}\,{\rm{curcumin}}/{\rm{Initial}}\,{\rm{amount}}\,{\rm{of}}\,{\rm{curcumin}}\,{\rm{loading}})\times 100 \% $$

#### Measurement of particle size, size distribution as well as zeta potential

Particle size, zeta potential as well as size distribution of curcumin liposomes was tested by Malvern Zetasizer Nano ZS90 (Malvern Instruments Ltd., Malvern, UK). The particle size distribution was considered as the polydispersity index (PDI).

#### Morphological observation of curcumin liposomes

Curcumin liposomes (2.5 µl) were added to a piece of glass (5 mm × 5 mm × 0.2 mm) and dried slowly with nitrogen, in order to get a homogeneous film. The liposomes were coated with gold in a sputter coater for 4–5 min and the morphological observation of gold-sprayed liposomes was carried out by SEM (FESEM S-4800, JEOL, Japan). Images were obtained from random positions of curcumin liposomes on the glass. Each measurement was done in triplicate.

#### Curcumin release

The release of curcumin from the liposomes was determined using equilibrium dialysis in PBS (0.01 M, pH 5.8) containing 30% ethanol (w/v). A total of 5 ml curcumin liposomes solution was added into a dialysis bag and then exposed to 15 ml release medium at 37 °C. The sample (1 ml) was withdrawn from the dialysis bag at predetermined time intervals and replaced with fresh release medium. The content of curcumin was measured by HPLC and calculated as cumulative percent released. The curcumin was taken as control. The release experiments were done in triplicate.

### QS inhibitory test

#### Detection of AHLs

The AHLs produced by *A*. *sobria* were detected by *C. violaceum* CV026 and *A. tumefaciens* A136. *C. violaceum* CV026 is the mini-Tn5 mutant of *C. violaceum* ATCC31532, which is unable to produce AHLs and violacein itself, but it is susceptible to different exogenous AHLs contained acyl side chains of 4–6 carbon atoms. *A. tumefaciens* A136, which carries a *lac*Z fusion to *tra*I, is able to produce a blue color in the presence of X-Gal and long chain AHLs. *C. violaceum* CV026 was grown in 10 ml of LB medium containing 10 μl kanamycin (20 μg/ml) at 28 °C, shaking at 160 rpm to an OD_600_ of 1.0. *A. tumefaciens* A136 was grown in 10 ml of LB medium containing 10 μl of tetracycline (20 μg/ml) and 50 μl of spectinomycin (20 μg/ml) at 28 °C, shaking at 160 rpm to an OD_600_ of 1.0. *C. violaceum* CV026 (1 ml) and *A. tumefaciens* A136 (1 ml) were added to 100 ml of nutrient agar, respectively. After the temperature of the medium dropped to room temperature, *A. sobria* was streaked on the two plates respectively to detect the presence of quorum sensing molecules. The *A. tumefaciens* A136 plates were supplemented with 100 μl of X-Gal (20 mg/ml).

#### Determination of MIC of curcumin liposomes and free curcumin

The MIC of curcumin liposomes and free curcumin against *C. violaceum* CV026, *A. tumefaciens* A136 and *A. sobria* respectively was measured by broth macrodilution method^27^. It was performed to ensure that the curcumin liposomes and free curcumin interfered with the QS system of bacteria instead of inhibiting their growth. An overnight culture of *C. violaceum* CV026 (1.0 OD at 600 nm), *A. tumefaciens* A136 (1.0 OD at 600 nm) and *A. sobria* (1.0 OD at 600 nm) were diluted 1:100 in 10 ml LB medium, respectively. To determine the MIC of curcumin liposomes and free curcumin, the medium was supplemented with twofold diluted free curcumin and curcumin liposomes respectively and incubated at 28 °C for 24 h. The absorbance of the medium at 600 nm was measured before and after incubation by spectrophotometer. The MIC was recorded as the lowest concentration, which exhibited complete inhibition of visible growth of test bacteria. The following anti-quorum sensing assays (biofilm formation, siderophore, extracellular protease, swimming and swarming motility as well as AHLs production) of curcumin liposomes and free curcumin were performed only at sub-MIC concentrations. Cholesterol, lecithin, 1% DMSO as well as the sterile water were served as blank control.

#### Violacein inhibition assay and β-galactosidase assay

The violacein inhibitory assay and β-galactosidase assay were performed according to the method of Issac^28^. Violacein inhibitory assay was tested by using *C. violaceum* CV026. Violacein production is dependent on the external addition of 10 μl of C6-HSL (20 μg/ml). In *A. tumefaciens* A136, the gene lacZ expressed and broke down X-Gal to produce blue color due to the addition of 10 μl of C8-HSL (20 μg/ml). Overnight culture of *C. violaceum* CV026 (1.0 OD at 600 nm) and *A. tumefaciens* A136 (1.0 OD at 600 nm) were diluted 1:100 in 10 ml LB medium, respectively. The diluted *C. violaceum* CV026 (1 ml) was grown at 28 °C on LB plates (20 ml) containing 1.5% agar and 20 μg/ml C6-AHL (10 μl). *A. tumefaciens* A136 (1 ml) was grown at 28 °C on LB plates (20 ml) containing 1.5% agar and 20 μg/ml C8-AHL (10 μl) as well as 100 μl of X-Gal (20 mg/ml). After the medium solidified, curcumin liposomes (100 μl) at sub-MIC concentration were added to the plates. Cholesterol (50 μl) and lecithin (50 μl) were mixed and served as blank control. The plates were incubated at 28 °C for 24 h and the reduction in violacein production and blue color was observed.

#### Growth curve analysis

In order to ensure that the curcumin liposomes affected the quorum sensing system of bacteria instead of interfering with bacterial growth, growth curve was measured in the presence of different concertrations of curcumin liposomes and free curcumin under sub-MIC, respectively. The determination method of growth curve was performed according to the previous literature^29^ with slight modification. Overnight culture (1%) of *A. sobria* (adjusted to 0.1 OD at 600 nm) and the curcumin liposomes under MIC were added to 10 ml LB medium. As control, the growth curve was obtained in the absence of curcumin liposomes. The solution of curcumin liposomes with fresh LB medium was taken as blank control to measure background curcumin liposomes level. The tubes were put in an incubator shaker at 30 °C 160 r/min for 24 h and the biomass of the *A. sobria* was measured by enzymatic analyzer (Perkin Elmer V3, USA) at 600 nm every four hours. The growth curve of *A. sobria* in the presence of free curcumin was measured in the same method.

#### Preparation of cell-free supernatant

Overnight culture of *A. sobria* (1.0 OD at 600 nm) treated or untreated with curcumin liposomes and free curcumin respectively was centrifuged at 12000 r/min for 15 min. The bacterial cells were discarded and the supernatant was filtered by using 0.22 μm sterilized film. The supernatant was stored at −20 °C for extracellular protease assay and siderophore production assay.

#### Determination of biofilm formation, SEM (Scanning electron microscope) observation and confocal laser scanning microscope (CLSM) observation

The qualitative determination of biofilm formation was performed following the method of Gordon Ramage *et al*.^30^ with some modifications. This method was performed to measure the effect of curcumin liposomes and free curcumin on biofilm formation of *A. sobria*, respectively. Briefly, the assay was carried out in 96 well flat bottom plastic microplates (Sarstedt Inc, USA). An overnight culture of *A. sobria* (grown in LB medium, 1.0 OD at 600 nm) was diluted 1:100 in fresh sterile LB medium. The dilution of *A. sobria* in the presence or absence of curcumin liposomes under sub-MIC was added to each well. The dilution of *A. sobria* with sterile water in the absence of curcumin liposomes was used as control. Curcumin liposomes with fresh LB medium was served as blank control to measure background curcumin liposomes level. The microtiter plate was incubated in at 28 °C for 48 h. Subsequently, the biofilm was washed with phosphate buffered saline (PBS, 7 mM Na_2_HPO_4_, 3 mM Na_2_H_2_PO_4_, and 130 mM NaCl at pH 7.4) for three times to remove loosely adherent cells. XTT was dissolved in PBS to obtain a final concentration of 0.5 mg/ml. This solution was filtered through a 0.22 μm filter and then stored at −70 °C. Menadione was dissolved in acetone (10 mM). Before the determination of biofilm formation, menadione was added to XTT to attain the final concentration of 1 μM. XTT-menadione solution (200 μl) was added to the wells and incubated in the dark at 37 °C for 2 h, and the absorbance at 490 nm was determined with a microplate reader (Bio-tek, Vermont, USA). The values were the mean of four measurements. The effect of curcumin on biofilm formation of *A. sobria* was performed in the same method.

An overnight culture of *A. sobria* (grown in LB medium, 1.0 OD at 600 nm) was diluted 1:100 in LB medium. The diluted culture (20 ml) of *A. sobria* was added to a petri dish. Then the curcumin liposomes were added to the petri dish to make the concentration of 420 μg/ml. As a control, the diluted culture of *A. sobria* was grown in the absence of curcumin liposomes. A piece of polished zinc (5 mm × 5 mm × 0.2 mm) was immersed in the above culture to allow the attachment of biofilm. The microtiter plate was statically incubated at 28 °C for 48 h. After incubation, the zinc was taken out and washed gently with sterile water and air dried for 30 min. Then the zinc was immersed into 2–5% (v/v) glutaraldehyde (precooling at 4 °C) for 4 hours and then washed with 50%, 70%, 80% and 90% (v/v) ethanol for 10 min respectively and 100% ethanol for 15 min for twice. Lastly, the zinc was immersed in 25% isoamyl acetate for twice, 15 min each and air dried. The morphological observation of the biofilms on the piece of zinc was performed by SEM after the biofilms were gold coating by a sputter coater. Images were obtained from random positions of biofilms. The SEM observation of biofilm treated with free curcumin was performed in the same method. Each measurement was done in triplicate.

A piece of above zinc attached with biofilms was washed with PBS for three times. Then, it was stained with 0.01% acridine orange (w/v, dissolved in PBS) for 15 min in the dark. After staining, it was washed with sterile PBS for three times and dried for 30 min. Antifade mounting medium (10 μl) was added to the biofilm, and then the biofilms were observed by CLSM (ZEISS, German) (emission: 525 nm, excitation: 488 nm). The 3-dimensional images of biofilms were obtained by ZEN Lite 2012 software (Carl Zeiss Microscopy GmbH, Germany).

#### Determination of extracellular protease

The determination of extracellular protease was performed following the method of Vijayaraghavan^31^. It was determined to elucidate the effect of curcumin liposomes on the QS-regulated production of extracellular protease of *A. sobria*. A transparent zone around the hole indicated the presence of extracellular protease activities. A total of 10 ml 1.5% (w/v) milk (115 °C, 0.06 MPa, 30 mim) was added to 90 ml nutrient agar when the temperature dropped to 50 °C. The above filtered cell-free supernatant (section 2.4.5) in the presence of curcumin liposomes (100 μl) was added to the well and sterilized water (100 μl) was served as the blank control. The extracellular protease was determined by measuring the diameter of the transparent zone around the hole in the milk plates. The effect of free curcumin on production of extracellular protease was performed in the same method. Each measurement was done in triplicate.

#### Determination of swimming and swarming motility

The effect of curcumin liposomes and free curcumin on the QS-regulated swimming and swarming motility in *A. sobria* was determined, respectively. Swimming medium contained 1 g tryptone, 0.5 g sodium chloride, 0.3 g agar and 100 ml deionized water. Swarming medium contained 1 g peptone, 0.5 g sodium chloride, 0.3 g agar, 0.5 g D-fructose and 100 ml deionized water. The medium were sterilized at 121 °C for 15 min and solidified at room temperature. Every swimming and swarming plate contained 20 ml medium. A total of 1.5 μl of overnight culture of *A. sobria* (grown in LB medium, 1.0 OD at 600 nm) supplemented with curcumin liposomes under sub-MIC was inoculated in the swimming and swarming plates, respectively. The control plates were prepared without curcumin liposomes. The plates were incubated at 28 °C for 24 h. The migration distance of *A. sobria* was measured. Every experiment was performed in triplicate.

#### Determination of AHLs production

Extraction of the AHLs. The AHLs produced by *A. sobria* in the presence and absence of curcumin liposomes and free curcumin was extracted, respectively. The overnight cultures of *A. sobria* treated with curcumin liposomes or free curcumin under sub-MIC were centrifugated at 12,000 g for 4 min respectively to obtain the cell-free supernatant. Then, the supernatant was extracted by ethyl acetate (containing 0.1% acetic acid) for twice. Then the supernatant was concentrated by rotary evaporation at 35 °C. The extract was re-dissolved by using methanol (1 ml) and filtered through 0.22 μm film for GC-MS (Gas-chromatography-mass-spectrometry) detection. As control, the overnight cultures of *A. sobria* were in the absence of curcumin liposomes and free curcumin, respectively.

The identification of the AHLs *via* GC-MS. Analyses of the AHLs were performed *via* a GC-MS Agilent 7890 N/5975 (Agilent, USA) following the method of Zhu *et al*.^32^ with slight modification. All sample injections were done in the split mode (50:1) into a HP-5 MS capillary column (30 m length × 0.25 mm internal inameter × 0.25 μm film thichness). Helium was used as the carrier gas at a flow rate of 1 ml/min. The GC injector temperature was 200 °C and the oven temperature was programmed as followed: 150 °C ramped at 10 °C/min to 220 °C, and ramped at 5 °C/min to 250 °C, then ramped at 0.5 °C/min to 252.5 °C. Mass spectrometry conditions were as followed: electron ionization source was set to 70 eV; MS Quad 150 °C, emission current 500 μA, MS Source 230 °C. Data were acquired by full-scan mode (m/z 35–800) and selected ion monitoring (SIM) mode (m/z 143).

#### Determination of siderophore production

The effect of curcumin liposomes and free curcumin on the QS-regulated siderophore production in *A. sobria* was determined on solid media using Chrome-Azurol-S CAS-agar, respectively. The color of the plates was blue due to the CAS (Chrome Azurol Sulphonate) complex, ferric iron and hexadecyltrimethylammonium (HDTMA). The color changed to orange when iron was removed by siderophores. Solution A: 0.07 g CAS was dissolved into 50 ml distilled water and 10 ml of 1 mmol/l FeCl_3_ was added (containing 10 mmol HCl); Solution B: 0.06 g HDTMA was dissolved into 40 ml distilled water; Solution C: Solution A was added to solution B slowly and mixed and the solution C was obtained. Then solution C was sterilized at 121 °C for 15 min. Subsequently, 0.2 ml of 1 mmol/l CaCl_2_, 0.2 ml of 1 mmol/l MgSO_4·_7H_2_O, 2 g agar and 6 ml of 10% (w/v) acid hydrolysis of casein (121 °C sterilized separately) together with Pipes (Sigma) (adjust pH 6.8–7.0) were diluted with water to 100 ml and sterilized at 121 °C for 15 min. Solution C (5 ml) was added to the above medium at 50 °C and mixed. Every plate contained 20 ml medium. The above mentioned filtered cell-free supernatant (section 2.4.5) in the presence of curcumin liposomes (100 μl) was added to CAS plates. The filtered cell-free supernatant without curcumin liposomes or free curcumin was served as control. The plates were incubated at 28 °C for 24 h. The relative amount of siderophores produced were determined by mesuring the diameters of orange halos.

### *In silico* analysis

#### Protein structure file and ligands

All calculations were performed on a LENOVO Z460 PC equipped with a double processor with the SYBYL-X 2.1 (Triops, Triops Inc., USA). The amino acids sequences of acyl-homoserine-lactone synthase and transcriptional regulators of *A. sobria* were obtained from the National Centre for Biotechnology Infromation (NCBI) (acyl-homoserine-lactone synthase, WP_042019484; transcriptional regulator, WP_042019486). The models of acyl-homoserine-lactone synthase and transcriptional regulators of *A. sobria* were built and assessed by using SWISS-MODEL^33, 34^ (https://swissmodel.expasy.org) and RAMPAGE (http://mordred.bioc.cam.ac.uk/~rapper/rampage.php), respectively. The most homologous sequence was used as template for modeling. A total of 50 models were built by SWISS-MODEL and the best model with the highest similarity and GMQE (Global Model Quality Estimation) score was selected. The models of acyl-homoserine-lactone synthase and transcriptional regulators of *A. sobria* were subjected for energy minimization by using the SYBYL-X 2.1 program. The 3D structure of curcumin, furfuran C-30 and C6-HSL were obtained from ZINC (http://zinc.docking.org/browse/subsets/) and fully geometry-optimized by using the standard Tripos molecular mechanics force field. The convergence criterion was set to be 0.005 kcal/(Å mol). Partial atomic charges were calculated through the Gasteiger-Hückel method. The structure of curcumin, furfuran C-30 and C6-HSL were converted into mol2 files for molecular docking.

#### Model assessment and molecular docking

The proteins models of acyl-homoserine-lactone synthase and transcriptional regulators *i.e*. LuxI and LuxR type proteins of *A. sobria* were assessed by using SWISS-MODEL Protein Structure & Model Assessment Tools and RAMPAGE. The docking analysis was performed to describe the interactions between the ligands and the modeled LuxI and LuxR type proteins of *A. sobria* by surflex-Dock in SYBYL-X 2.1 program. The two built proteins were analyzed and prepared by SYBYL-X 2.1 and the water molecules in the models were removed. The docking boxes of two built proteins of *A. sobria* were chosen to be a radius of 5 Å around the ligands, and the residues which were not in the active site were hided. The mol2 files of curcumin, furfuran C-30 and C6-HSL were docked with the built models and the best conformation of each ligand could be obtained.

### Statistical analysis

The results were expressed as means standard deviation (SD). Analysis of variance was conducted and differences between variables were tested for significance by one-way ANOVA with Tukey test using the SPSS Statistics 20.0, Origin Pro 9.0; Differences at P < 0.05 were considered statistically significant. All the experimental data represented the mean of triplicate values.

### Data availability statement

All data are fully available without restriction.

## Results and Discussion

### SEM analysis and characteristics of curcumin liposomes

**Figure 1:**
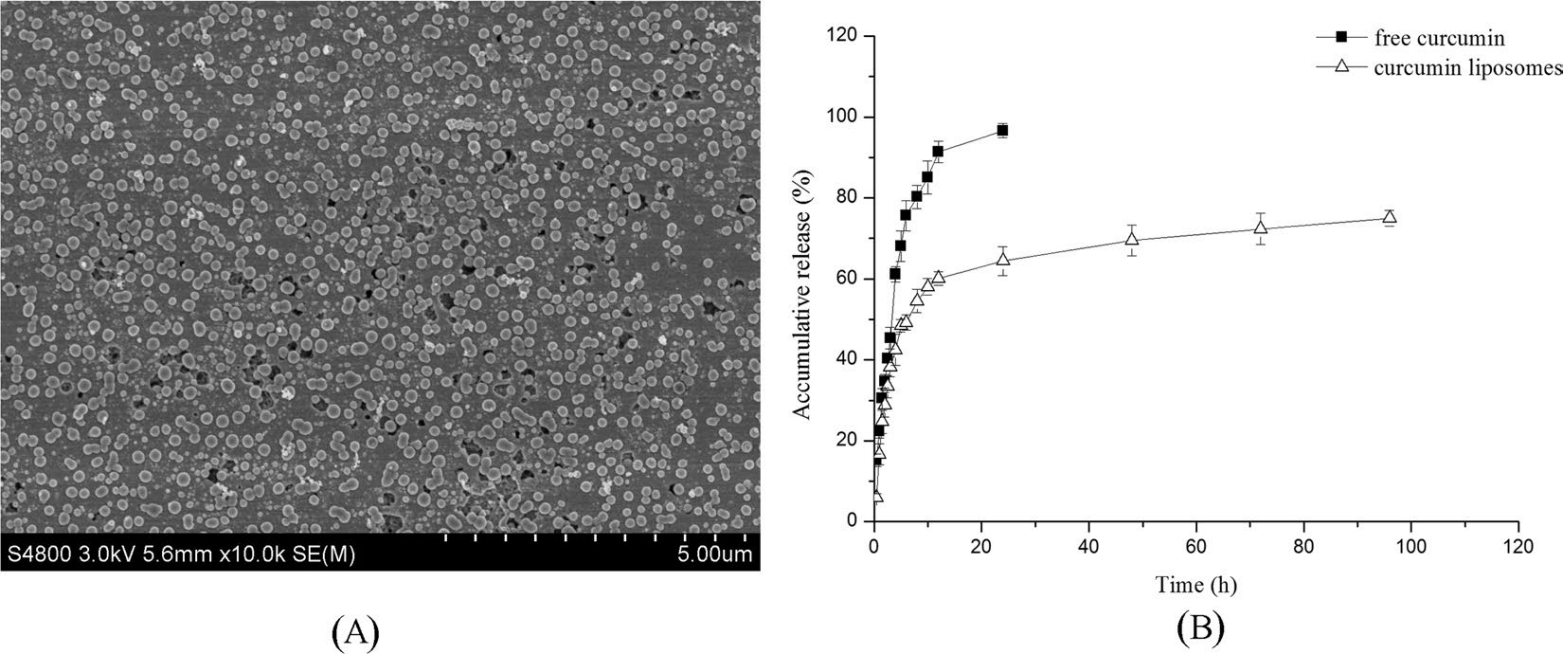
SEM image (**A**) and curcumin release (**B**) of prepared curcumin liposomes.

The SEM image of curcumin liposomes was shown in Fig. 1(A), the curcumin liposomes were in spherical or ellipsoidal shapes and distributed uniformly. The characteristics including particle size, zeta potential, PDI value, encapsulation efficiency and loading capacity were then measured. As listed in Table 1, the curcumin liposomes were similar in size (see Supplemental Fig. S1) and the encapsulation efficiency was high (84.51 ± 0.58%). The liposomes were further stored in 4 °C and their particle size and encapsulation efficiency were measured every two days for two weeks. The results showed that, the particle size and encapsulation efficiency varied very little, confirming that the curcumin liposomes were very stable. Chen *et al*. had prepared different curcumin-loaded liposomes by using soybean phospholipids, hydrogenated soybean phospholipids and egg yolk phospholipids. The highest encapsulation efficiency of these liposomes was 82.32 ± 3.91%, which was lower than that of our study^17^. The results indicated that liposomes were excellent carriers for curcumin. Basak *et al*. also used liposomes to pack curcumin-difluorinated to treat head and neck squamous cell carcinoma^35^. Narayanan *et al*. used the liposome to encapsulate curcumin and resveratrol and they found that, the two drugs in combination had the ability to reduce prostate cancer incidence in PTEN knockout mice^36^. Similarly, Agashe and coworkers used liposomes to package EF24-HPβCD and they found that, the drugs had an anti-proliferative effect in lung and prostate cancer cell lines^37^. Therefore, it was practicable to use liposomes to package curcumin to improve the its bioavailability.

**Table 1:** Characteristics of curcumin liposomes.

Characteristics	Particle size (nm)	Zeta potential (mV)	PDI	Encapsulation efficiency (%)	Loading capacity (%)
Value	159.3 ± 6.8	−39 ± 2.70	0.156 ± 0.003	84.51 ± 0.58	2.87 ± 0.02

The release of curcumin from the curcumin liposomes was then examined. As shown in Fig. 1(B), the curcumin liposomes revealed a sustained-release trend. 34.65% of curcumin was released from the release medium, but only 28.87% of curcumin was released from curcumin liposomes within the first 2 h. 96.65% of the curcumin was released in medium, while only 64.39% of curcumin was released from curcumin liposomes after 24 h. The figure showed that, curcumin released quickly from curcumin liposomes at first and then given a slower release, resulting in a higher drug concentration at first, and then slow release of the remaining curcumin maintained efficacy for a longer time.

### MIC of curcumin liposomes and free curcumin

Since curcumin has shown better antimicrobial activities, MIC should be measured to ensure that the inhibitory effect was due to interfering with the QS system of bacteria rather than inhibiting their growth. MIC of curcumin liposomes or free curcumin was the lowest concentration that exhibited complete inhibition of visible growth of *C. violaceum* CV026, *A. tumefaciens* A136 and *A. sobria*. The MIC of curcumin liposomes against *C. violaceum* CV026, *A. tumefaciens* A136 and *A. sobria* were 400 µg/ml, 460 µg/ml and 420 µg/ml, respectively. The MIC of free curcumin against *C. violaceum* CV026, *A. tumefaciens* A136 and *A. sobria* were 200 µg/ml, 370 µg/ml, and 280 µg/ml, respectively. All further studies were carried out at sub-MIC concentrations.

### Violacein inhibition test and β-galactosidase assay

**Figure 2:**
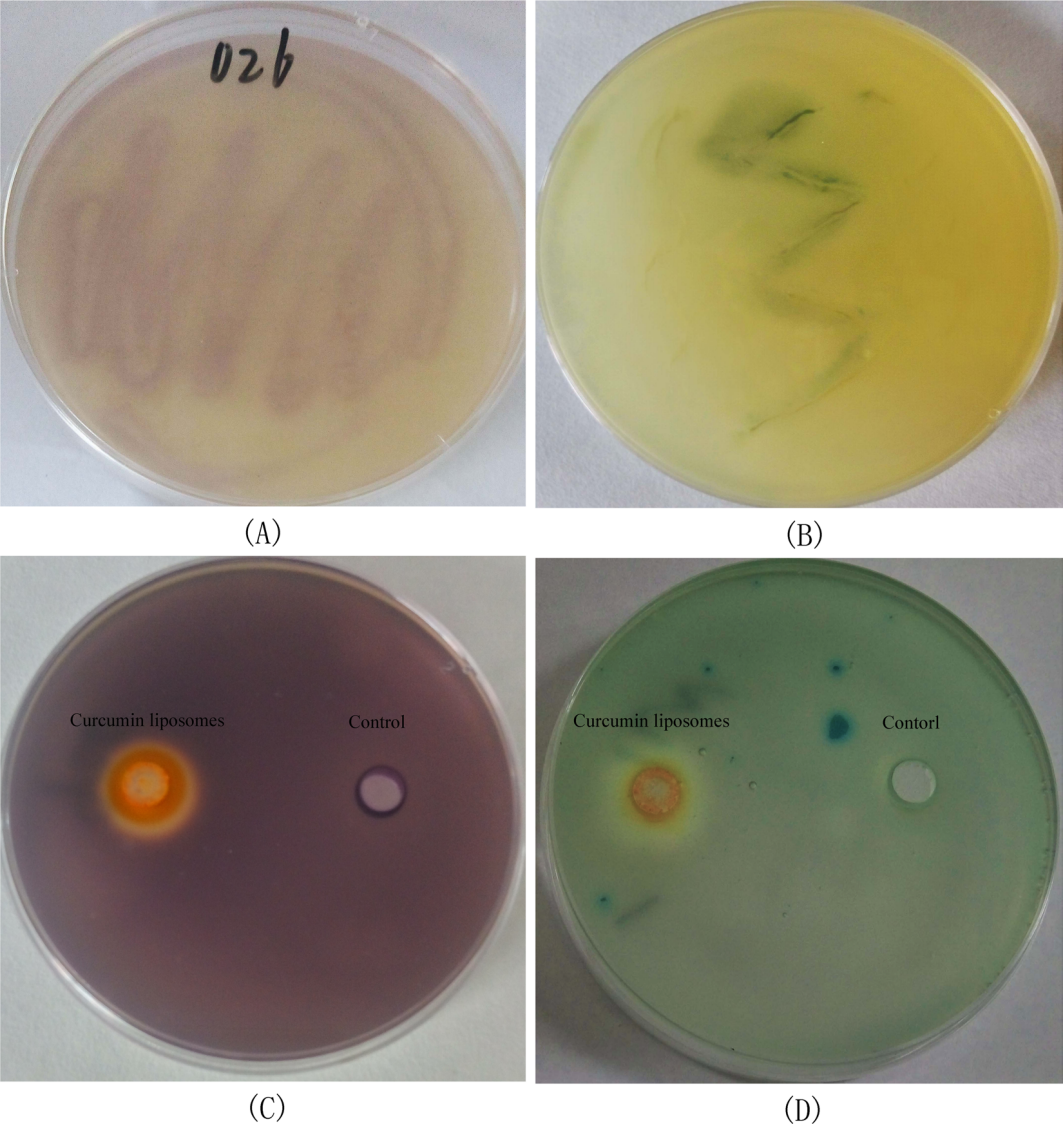
Detection of AHLs produced by *A. sobria via* indicator bacteria of *C. violaceum* CV026 (**A**) and *A. tumefaciens* A136 (**B**) as well as violacein inhibition assay (**C**) and β-galactosidase assay (**D**) of curcumin liposomes.

The detection of QSIs was largely facilitated by the application of biosensors, which allow rapid screening of substances with QS inhibitory activities. As shown in Fig. 2(A,B), AHLs produced by *A. sobria* were detected by *C. violaceum* CV026 and *A. tumefaciens* A136. Result showed that, *A. sobria* produced both short and long chain AHLs. In Fig. 2(C), the color of control was purple, and this was because of the expression of CviR (a luxR homologue) by acceptance of C6-HSL in the plate^38^. While there was loss of purple pigment instead of transparent and clear zone around the hole treated with curcumin liposomes. In Fig. 2(D), there was halo zone around the hole in the presence of curcumin liposomes, while the color of the control remains blue. The result indicated that, the curcumin liposomes had exhibited inhibitory effect on the QS system of bacteria rather than affecting the bacterial growth.

### Effects of curcumin liposomes on swimming and swarming motility, siderophore as well as extracellaluar proteases of *A. sobria*

**Figure 3:**
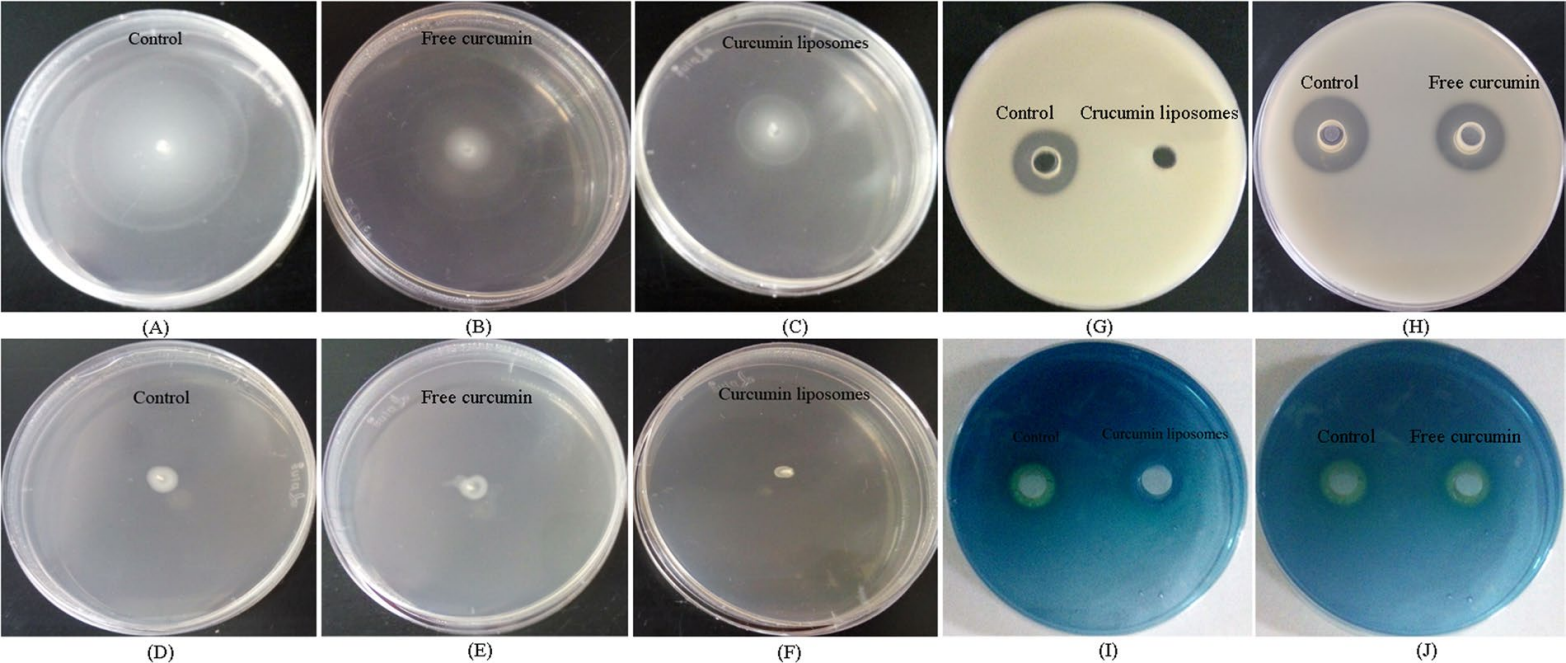
Inhibitory effect of curcumin liposomes and free curcumin (420 µg/ml of liposomes and 280 µg/ml of free curcumin) on swimming (**A**–**C**) and swarming motility (**D**–**F**), extracellaluar proteases (**G**,**H**) as well as siderophore (**I**,**J**) of *A. sobria*.

Bacteria mainly relied on flagella in their motility, which was closely related to biofilm formation. Swarming was a group action, while swimming was individual behavior. Both the two bacterial motilities were controlled by QS system, by which bacteria could evade antibacterial agents and body’s immune system^39^. Many bacteria have shown swimming and swarming motility like *Proteusbacillus vulgaris*, *Vibrio* spp., *Bacillus* spp., *Salmonella* spp. and *Serratia marcescens*. As could be seen from Fig. 3 (A–F), the strain of *A. sobria* migrated from inoculation zone and formed round colonies in swimming and swarming plates. Curcumin liposomes and free curcumin showed a considerable reduction in the motility of *A. sobria* when compared with control, indicating that the QS-controlled swimming and swarming motility of *A. sobria* was significantly inhibited. Additionally, curcumin liposomes were more effective than free curcumin in inhibiting swimming and swarming motility of *A. sobria*. Similarly, Kazemian and coworkers have found that, the extract of *Chamaemelum nobile* has shown inhibitory effect on the QS-regulated swarming motility and biofilm formation of *Pseudomonas aeruginosa* ^40^.

Spoilage microorganisms decompose proteins in food into small molecular peptides and amino acids by secreting extracellular protease, and ultimately produce volatile and stimulating nitrogen- and sulfur-containing compounds *via* a series of bacterial metabolisms, resulting in the spoilage of food. Extracellular protease was also one of the most important QS-regulated virulence factors in many bacteria. Christensen *et al*. studied the expression of QS-controlled extracellular protease in *Serratia proteamaculans* and they found that, the AHL (3-oxo-C6-HSL) had a very large impact on exoenzymes activies^41^. In the skimmed milk plates, there was a large and transparent zone around the hole which was in the absence of curcumin liposomes, while the size of transparent zone around the hole treated with free curcumin was smaller than that of control. Further, there was no significant change around the hole in the present of curcumin liposomes (Fig. 3(G,H)). The results indicated that, the inhibitory effect of curcumin liposomes in inhibiting the QS-regulated secretion of extracellular protease was better than that of free curcumin.

Siderophore generally refers to a series of small molecular chelates (1–2 kDa) with strong and specific affinity to Fe^3+^, which is synthesized by microorganisms through non-ribosome pathway in low iron condition. The CAS plate was blue due to the ternary complex (CAS, HDTMA and Fe^3+^), but a faint yellow halo appeared around the hole when there were siderophore produced by bacteria. As shown in Fig. 3(I,J), there was no faint yellow halo around the hole in the presence of curcumin liposomes, which was contrary to that of control, suggesting that the QS-regulated siderophore production in *A. sobria* was inhibited by curcumin liposomes. The inhibitory effect of curcumin in siderophore production in *A. sobria* was not significant.

### Effects of curcumin liposomes on the growth curve and biofilm formation of *A. sobria*

Biofilms are aggregation of one or several species of bacteria, which are embedded in a extracellular polymeric substance (EPS)^13, 40^. EPS consists of extracellular DNA, polysaccharides and proteins, *etc*. Biofilms can attach to various surfaces, including food, medical devices, and host tissues^42, 43^. And once it forms, it is difficult to be removed even use modern technologies, causing great damages to human health and safety^44, 45^. Recent studies have found that the formation of biofilm was regulated by QS system^42^. Chan *et al*. have found that *Aeromonas veronii biovar sobria* strain 159 showed QS activities mediated by N-acyl homoserine lactone^46^. Suntharalingam *et al*. have found that the biofilm formation of *Streptococci* was associated with their QS system^47^. Similarly, Birgit Huber and coworkers^48^ found that, the biofilm formation and swarming motility of *Burkholderia cepacia* were regulated by its QS system. Thus, it is a feasible and potential way to hinder the bacterial biofilm formation by blocking their quorum sensing pathways.

**Figure 4:**
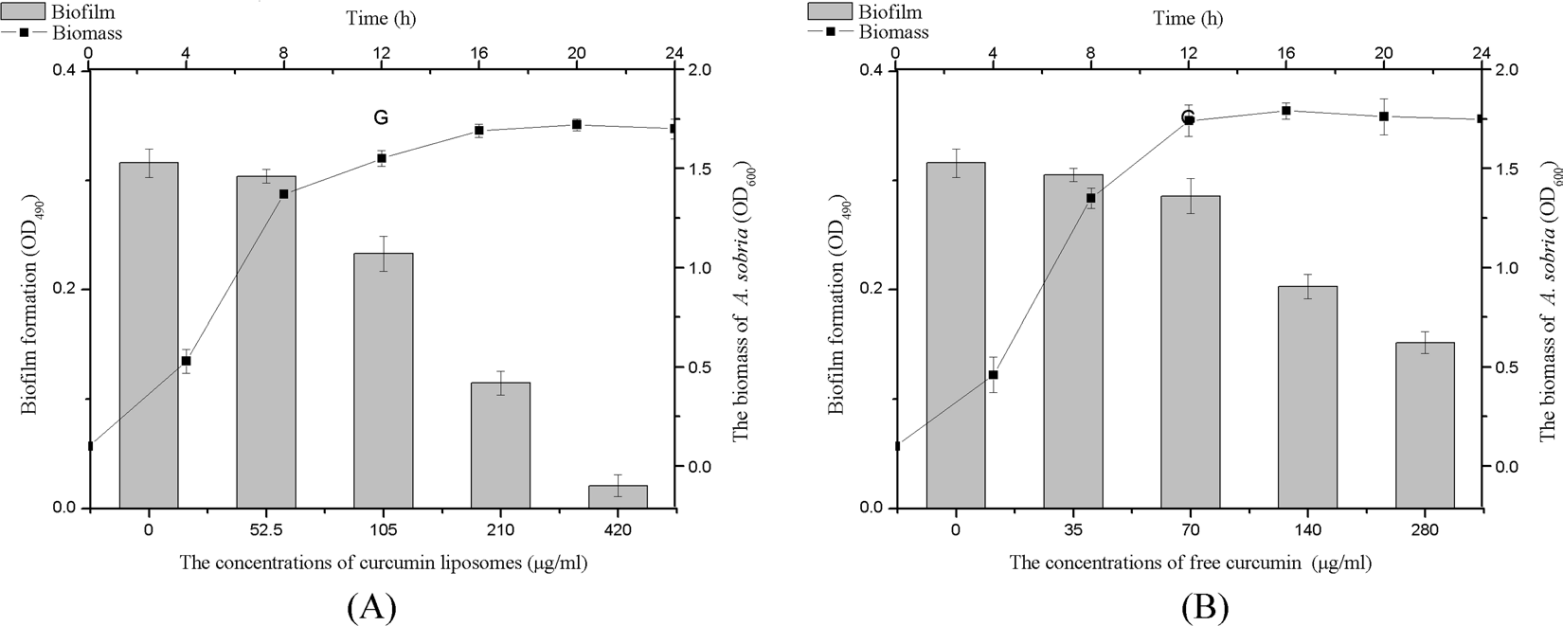
Effect of different concentrations of curcumin liposomes (1) or free curcumin (2) on growth and biofilm formation of *A. sobria*. Each bar and line represents mean of three replicates and standard deviations.

In qualitative analysis, the biofilm formation of *A. sobria* was inhibited by free curcumin and curcumin liposomes under sub-MIC in a concentration-dependent manner. The biofilm has fallen by 51.90% when treated with free curcumin at 280 µg/ml, whereas, curcumin liposomes inhibited biofilm formation up to 93.35% when compared to control (p < 0.05) at 420 µg/ml. More importantly, the growth curve of *A. sobria* was typical microbial growth curve (Fig. 4), indicating that the curcumin liposomes did not affect the growth of *A. sobria* but interfered with their QS system.

**Figure 5:**
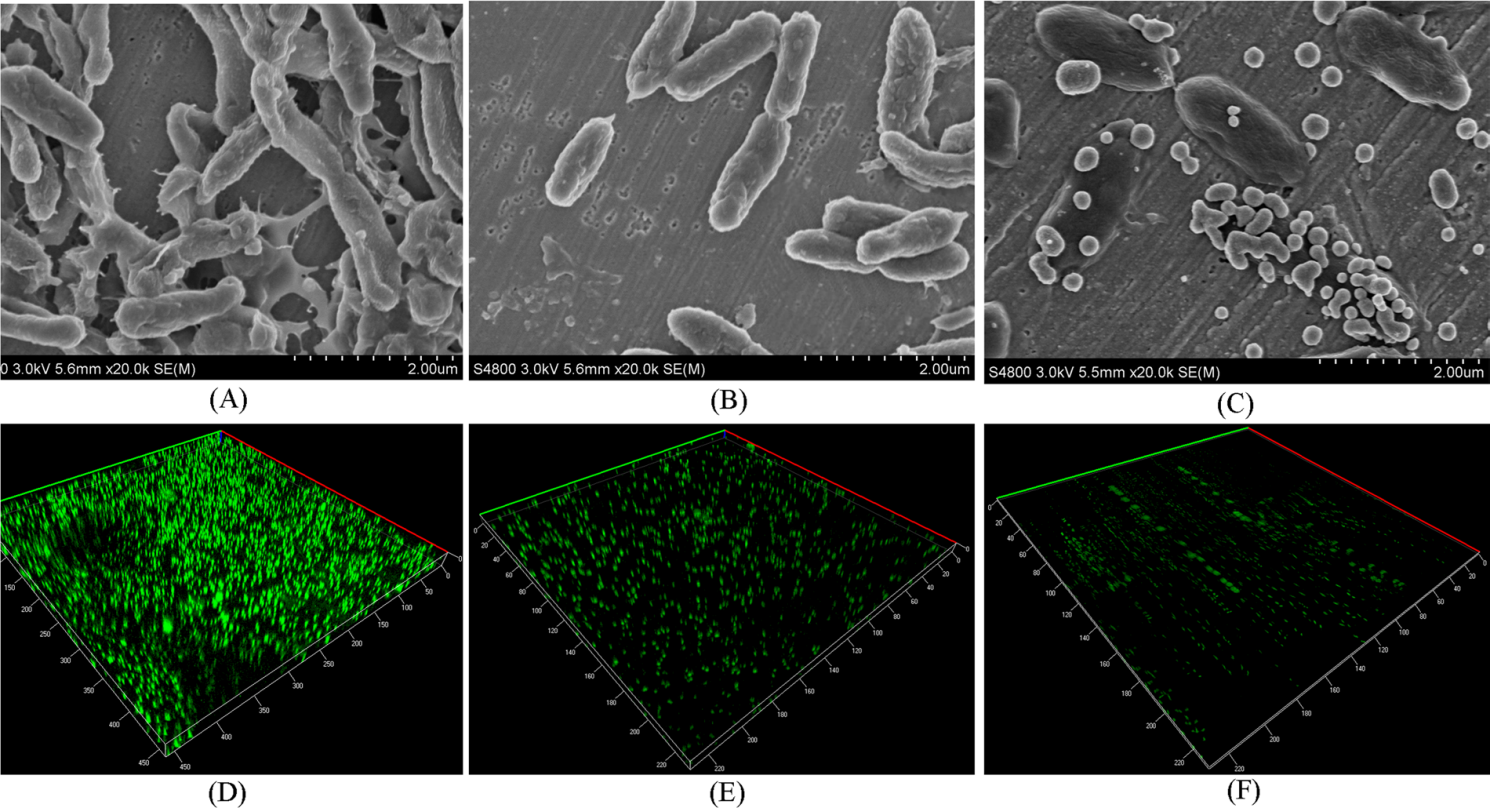
SEM analysis (**A**–**C**) and CLSM observation (**D**–**F**) of biofilm of *A. sobria* treated with curcumin liposomes and free curcumin. (**A**,**D**): biofilm of *A. sobria*; (**B**,**E**) biofilm of *A. sobria* treated with free curcumin; (**C**,**F**): biofilm of *A. sobria* treated with curcumin liposomes.

The biofilm produced by *A. sobria* could be observed clearly by SEM. The bacterial cells were rod-shaped, aggregated, and formed dense biofilm. Conversely, the biofilm was looser and bacterial cells were scattered when treated with free curcumin and curcumin liposomes. Little biofilms were formed when treated with curcumin liposomes (Fig. 5(A–C)). What’s more, the morphology of *A. sobria* was normal. The result also suggested that, the curcumin liposomes had inhibitory effect on the biofilm formation of *A. sobria* and they did not interfere with normal growth of bacteria. Qu Lin *et al*. got the similar conclusion that norspermidine could prevent and eradicate the biofilm formation of *P. aeruginosa*. And they also observed the initial attachment of biofilm by SEM^44^. QS-regulated production of EPS contributes the mature of biofilm^49^. From SEM images, EPS of biofilms treated with curcumin liposomes decreased significantly when compared with that of free curcumin and control. The reason may be that, the curcumin released from curcumin liposomes penetrated into bacterial cells due to the reduction of EPS and interfered with the QS system of bacteria. Junli Zhu *et al*. also found that, green tea polyphenols had inhibitory effect on the EPS production of *Shewanella balitica* ^50^. Interestingly, some of the curcumin liposomes were attached to the surface of bacterial cells. CLSM images (Fig. 5(D–F)) showed that, the color of control was very green and the biofilm was thick, dense, and compact, whereas the biofilm treated with free curcumin was dispersed and the intensity of green color was lower. The biofilm treated with curcumin liposomes was the thinnest and the green color was the lowest. The results revealed that curcumin liposomes significantly decreased the biofilm formation in *A. sobria*. The result was corresponded to the qualitative analysis.

### Effects of curcumin liposomes on AHL production of *A. sobria*

AHLs were the signal molecules used by most Gram-negative bacteria, and the AHLs might be different in N-acyl chain and carbon chain produced by various bacterial species^51^. Numerous studies have shown that AHLs-mediated quorum sensing was closely associated with bacterial pathogenicity^25, 45^.

It has been more accurate to detect and analyze AHLs quantitatively by using GC-MS (see Supplemental Fig. S2). Table 2 was the changes in AHLs production of *A. sobria* in the presence and absence of free curcumin and curcumin liposomes. *A. sobria* produced C4-HSL, C6-HSL, C10-HSL, C12-HSL and C14-HSL, while it produced C4-HSL, C6-HSL, C10-HSL and C14-HSL when treated with free curcumin. Only C4-HSL and C14-HSL produced when treated with curcumin liposomes under sub-MIC. C4-HSL, C6-HSL, C10-HSL and C14-HSL treated with free curcumin decreased by 64.65%, 37.67%, 83.60% and 73.00%, respectively. While C4-HSL and C4-HSL decreased by 71.36% and 93.55% respectively when treated with curcumin liposomes. The result showed that, the inhibitory effect of curcumin liposomes on AHLs production of *A. sobria* was more significant than that of free curcumin. This study was consistent with Luciardi *et al*. that the volatile metabolites of *Merremia dissecta* creeper had the ability to hinder the QS system of *Pseudomonas aeruginosa*. And the AHLs content decreased 63–75% by GC-MS detection^52^.

**Table 2:** Effect of curcumin liposomes and free curcumin on AHLs production of *A. sobria*.

Signal molecules	Peak time	Peak Area		
		Control	Treated with free curcumin	Treated with curcumin liposomes
C4-HSL	4.431	4,766	1,685	1,365
C6- HSL	6.286	4,959	3,091	—
C8- HSL	8.333	—	—	—
C10- HSL	10.720	76,520	12,550	—
C12- HSL	13.422	37,501	—	—
C14- HSL	16.939	99,515	26,862	6,419

–: Not detected.

### Sequence alignment and homology modeling

For a variety of difficulties, the structures of certain proteins could not be known, therefore, plenty methods were utilized to solve this problem including homology modeling. Computer homology modeling techniques are generally used to build three dimensional (3D) proteins with unknown structures. The 3D structures of LuxI and LuxR type proteins of *A. sobria* were built *via* sequence alignment and homology modeling by SWISS-MODEL. The models were built through Blast and HHBlits search to match the target amino acid sequence in the SWISS-MODEL template library. Plenty sequences were searched by SWISS-MODEL and the templates which similarity above 30% were selected to build models. Three and five models for LuxI and LuxR type protein respectively were selected based on the sequence similarities and GMQE score.

**Figure 6:**
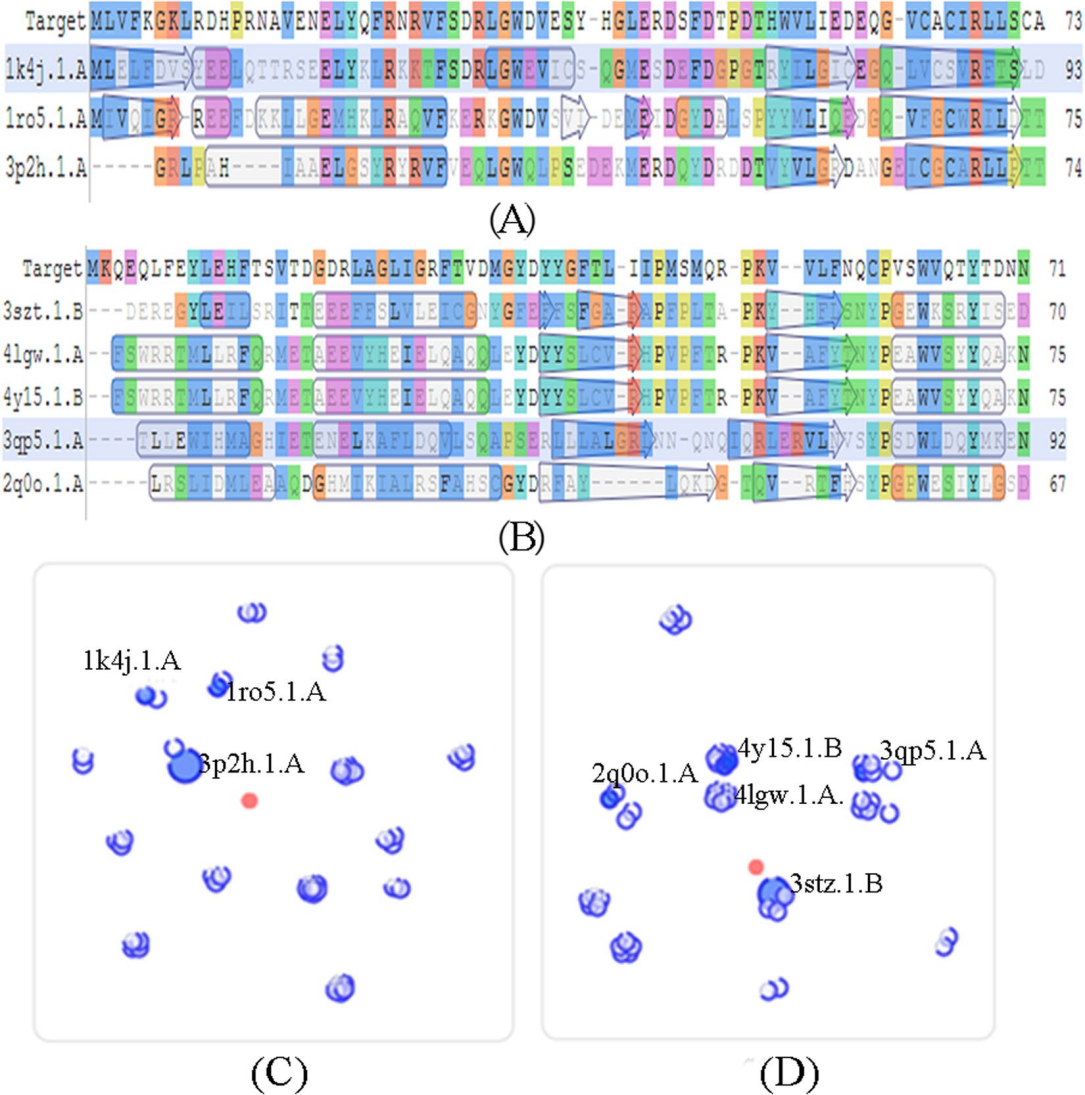
Part of sequence alignment results of LuxI (1) and LuxR type (2) proteins of *A. sobria* and their plots of scores (3: LuxI; 4: LuxR).

The sequence alignment results were shown in Fig. 6(A,B). The sequence alignment of the target protein with template proteins exhibited that, the residues in the active site were strictly conserved. There were three matches for LuxI type protein (acyl-homoserinelactone synthase EsaI, autoinducer synthesis protein lasI and AHL synthase) and five matches for LuxR type protein (transcriptional regulator of ftsQAZ gene cluster, regulatory protein SdiA, probable transcriptional activator protein traR, CviR transcriptional regulator and quorum-sensing control repressor) of *A. sobria* to build models (Table 3).

**Table 3:** Structures and scores of LuxI and LuxR type proteins models of *A. sobria*.

Proteins	Template	Description	GMQE	QMEAN	Model
LuxI type	1k4j.1.A	acyl-homoserinelactone synthase EsaI	0.54	−4.55	
	1ro5.1.A	autoinducer synthesis protein lasI	0.55	−2.28	
	3p2h.1.A	AHL synthase	0.61	−3.14	
LuxR type	4y15.1.B	transcriptional regulator of ftsQAZ gene cluster	0.63	−3.36	
	4lgw.1.A	regulatory protein SdiA	0.64	−2.91	
	2q0o.1.A	probable transcriptional activator protein traR	0.60	−3.88	
	3qp5.1.A	CviR transcriptional regulator	0.62	−4.85	
	3stz.1.B	quorum-sensing control repressor	0.63	−2.54	

The 3D models of LuxI and LuxR type proteins were built from the most homologous sequence of each protein. The AHL synthase (3p2h.1.A) and quorum-sensing control repressor (3stz.1.B) were selected as the templates for LuxI and LuxR type proteins modeling respectively based on the GMQE and QMEAN score^53^. The plots of scores of the proteins (Fig. 6(C,D)) also showed that, the 3p2h.1.A and 3stz.1.B for LuxI and LuxR type proteins respectively were nearest to the center points (target sequence), suggesting that the two models were the most suitable templates for builting LuxI and LuxR type proteins of *A. sobria*. The similarity of the templates was all above 30%, indicating that the build models could make a good prediction of the target proteins.

### Model quality assessment

**Figure 7:**
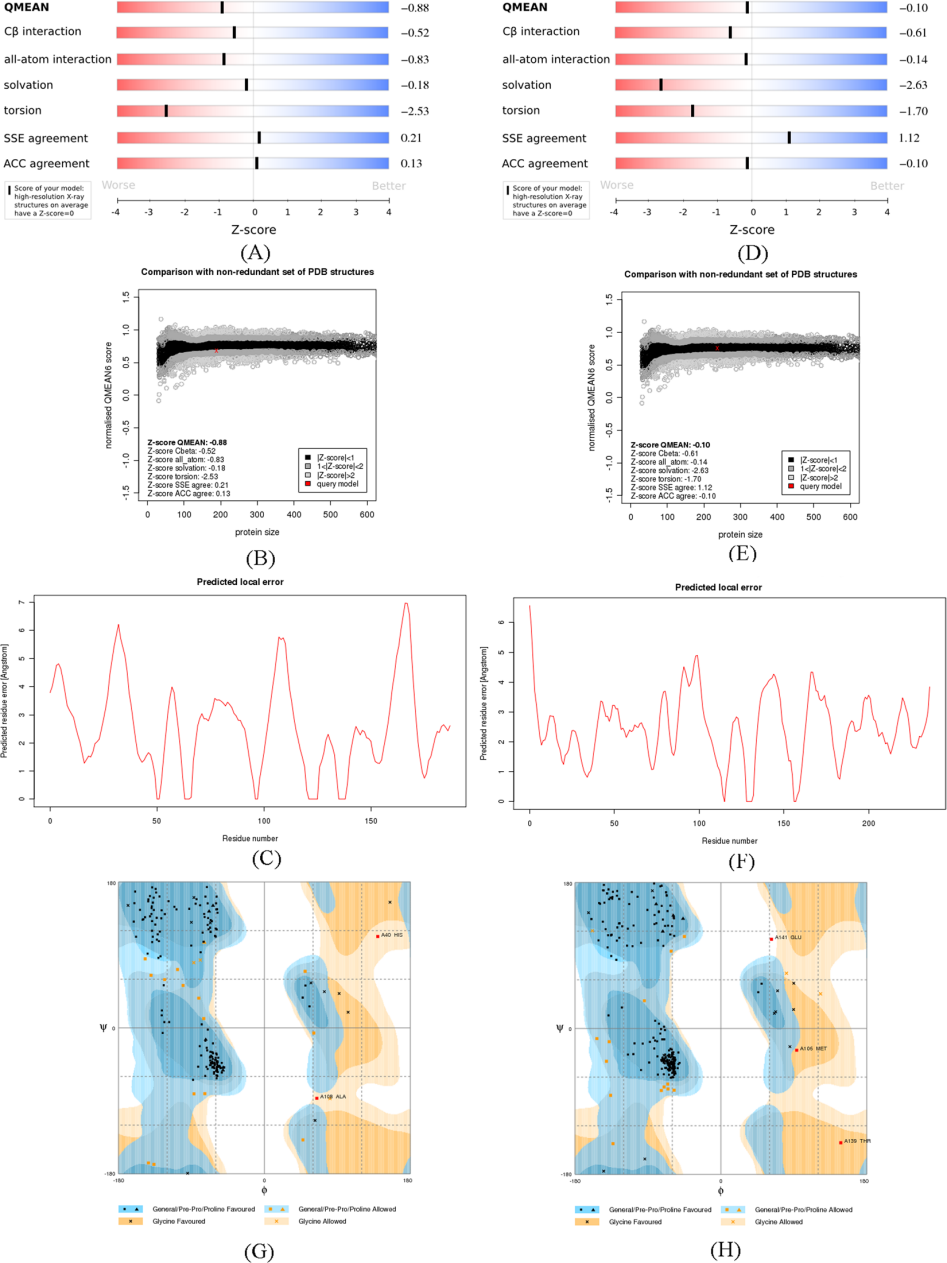
Assessment of the quality of LuxI (1-3) and LuxR (4–6) type proteins of *A. sobria* by SWISS-MODEL and RAMPAGE (7: Ramachandran Plot Analysis of LuxI type protein; 8: Ramachandran Plot Analysis of LuxR type protein).

The quality of build models were evaluated by using the SWISS-MODEL and RAMPAGE. QMEAN is a composite scoring function for both the estimation of the global quality of the entire model as well as for the local per-residue analysis of different regions within a model^53^. The QMEAN score and Z-score were 0.68 and −0.88 for LuxI type protein, and 0.76 and −0.10 for LuxR type protein of *A. sobria* (Fig. 7(A–F)). The results indicated that the models are valid, and the built models could make a good prediction for the 3D structures of LuxI and LuxR type proteins. The Ramachandran plot analysis (Fig. 7(G,H)) showed that, in LuxI type models, 89.1% residues were in favoured region, 9.8% in allowed region, 1.1% in the outlier region; whereas the corresponding values for the Lux R type protein were 91.7%, 7.0%, 1.3%, respectively. This ensured that the quality of generated model was good. Similarly, Al-Khayyat *et al*. have built the LuxI protein model, which was similar to the closely-related known structures by homology modeling method. And model 4 with the QMEAN6 score of 0.732 had better quality among the five built models and they selected ten compounds for docking analysis with the built models^54^.

### Docking analysis

Molecular docking analysis was performed to elucidate the mechanism of curcumin acted on the QS system of *A. sobria*. In order to clarify the interactions between curcumin and the two built proteins of *A. sobria*, curcumin was docked with the active sites of built LuxI and LuxR type proteins. Futher, C6-HSL was used as native ligand when docked with LuxR type protein. Furanone C-30 and its derivate *i.e*. furanone-56 were known inhibitors of QS system, which could interfere with the combination of AHLs with their receptor proteins. It has been reported to inhibit the production of biofilm and virulence factors as well as other physiological activities of *Pseudomonas aeruginosa* ^55^. Thus, furanone C-30 was used as positive control to be docked with LuxR type protein of *A. sobria*.

**Figure 8:**
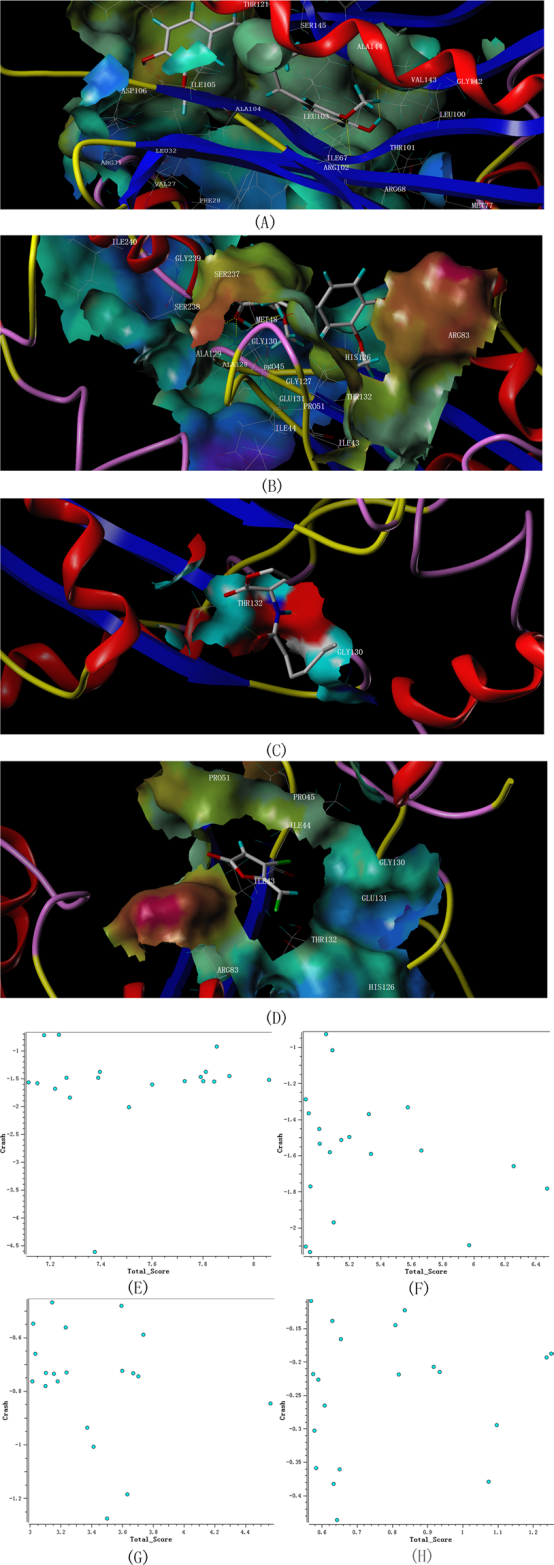
kGraphical representation of LuxI and LuxR type proteins of *A. sobria* docked with curcumin, C6-HSL and furanone C-30 as well as the conformations of the ligands. Yellow dashed line represents the Hydrogen-bonds. Residues in the active site were labeled. (1) Curcumin docked with modeled LuxI type protein (2) Curcumin docked with modeled LuxR type protein (3) C6-HSL docked with modeled LuxR type protein (4) Furanone C-30 docked with modeled LuxR type protein (5) Conformations of curcumin docked with LuxI type protein (6) Conformations of curcumin docked with LuxR type protein (7) Conformations of C6-HSL docked with LuxR type protein (8) Conformations of furanone C-30 docked with LuxR type protein.

Docking results of LuxI and LuxR proteins with their ligands were listed in Table 4. The results showed that, curcumin docked with the active site of LuxI and LuxR type proteins of *A. sobria* with total scores of 8.0610 and 6.4729, respectively. In addition, C6-HSL and furanone C-30 were also docked with Lux R type protein of *A. sobria* with total scores of 4.5625 and 1.2519, respectively. Molecular docking studies revealed that, curcumin showed higher *in silico* binding affinity to the LuxI type protein of *A. sobria*. The docking calculations with 20 conformations for each ligand (Fig. 8(E–H)) showed that, the conformations distribution of curcumin with LuxI type protein were more centralized than that of others. The above results suggested that the inhibitory mechanism of curcumin on QS system of *A. sobria* might be that, the curcumin combined with LuxI type protein and blocked the production of AHLs in *A. sobria*.

**Table 4:** Docking results of LuxI and LuxR type proteins of *A. sobria* with curcumin, C6-HSL and furanone C-30.

Protein	Ligand	Total score	Crash	Polar	Compound structure
LuxI type	curcumin	8.0610	−1.5280	3.7959	
LuxR type	curcumin	6.4729	−1.7836	2.0901	
	C6-HSL	4.5625	−0.8459	1.9111	
	furanone C-30	1.2519	−0.1880	0.0000

The interactions between the ligands and the two built models of *A. sobria* were analyzed by SYBYL-X 2.1. As shown in Fig. 8(A–D), curcumin interacted with key residues (VAL143, ARG102, LEU103, SER145 and ILE105) of LuxI type protein *via* hydrogen-bonding interactions. Similarly, the residues of LuxR type protein of *A. sobria* interacted with curcumin were found to be THR132, GLY130, ALA129 and GLY127. For the native ligand, C6-HSL has formed hydrogen bonds with GLY130 and THR132 residues in LuxR type protein, while there were no hydrogen bonds formed between furanone C-30 and LuxR type protein of *A. sobria*. Overall, the above results had demonstrated that, the QS system inhibited by curcumin liposomes was largely because that, the curcumin released from curcumin liposomes interacted with LuxI type protein by forming hydrogen bonds in *A. sobria*.

## Conclusion

This study exhibited that the prepared curcumin liposomes had significantly inhibited the QS-controlled siderophore production, swimming and swarming motility, extracellular proteases, biofilm formation and AHLs production of *A. sobria*. *In silico* molecular docking analysis revealed that, the QS system inhibited by curcumin liposomes was largely because that, the curcumin released from curcumin liposomes interacted with LuxI type protein by forming hydrogen bonds, which hindered the production of AHLs in *A. sobria*. The built LuxI and LuxR proteins might be useful and potential targets to design novel quorum sensing inhibitors.

## Electronic supplementary material

Supplementary Information
