# Supplementary material for: Curcumin liposomes interfere with quorum sensing system of Aeromonas sobria and in silico analysis
Source: Sci Rep. 2017 Aug 17;7:8612. doi: 10.1038/s41598-017-08986-9 (PMC5561023; doi:10.1038/s41598-017-08986-9)
Supplement: Supplementary file 1 — Supplementary Information [file 41598_2017_8986_MOESM1_ESM.pdf]

Curcumin liposomes interfere with quorum sensing system of *Aeromonas sobria and in silico* analysis

Ting Ding[a, c], Tingting Li[b], Zhi Wang[d], Jianrong Li[c, a*]

Fig.S1 Particle size (a) and zeta potential (b) of curcumin liposomes

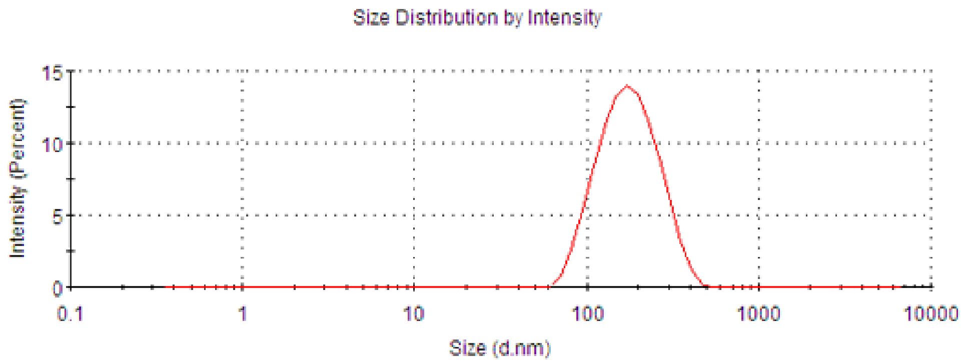

(a)

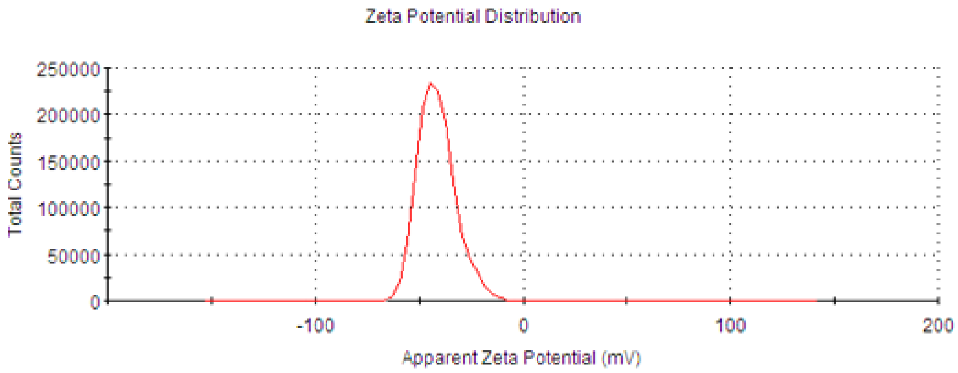

(b)

Fig. S2 Effects of curcumin liposomes and free curcumin on AHLs production of *A. sobria* (A) Typical GC-MS chromatogram of standard AHLs (B) GC-MS chromatogram of AHLs of *A. sobria* (C) GC-MS chromatogram of AHLs of *A. sobria* treated with free curcumin (D) GC-MS chromatogram of AHLs of *A. sobria* treated with curcumin liposomes

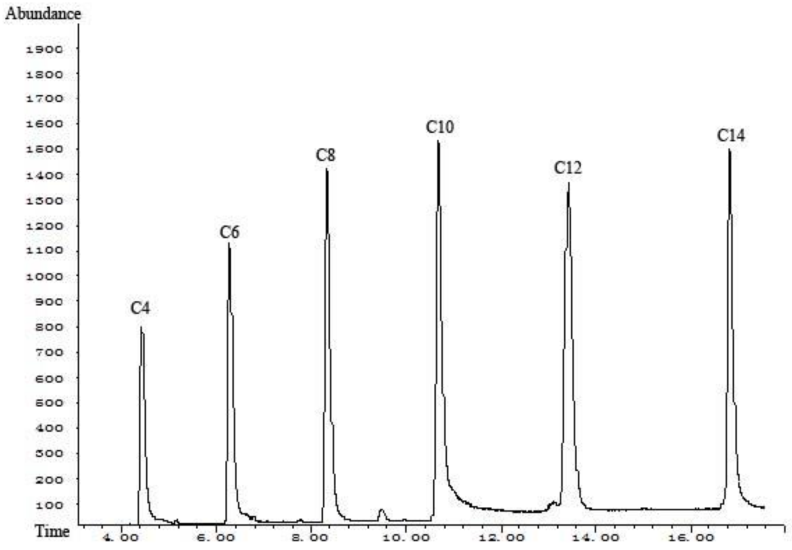

(A)

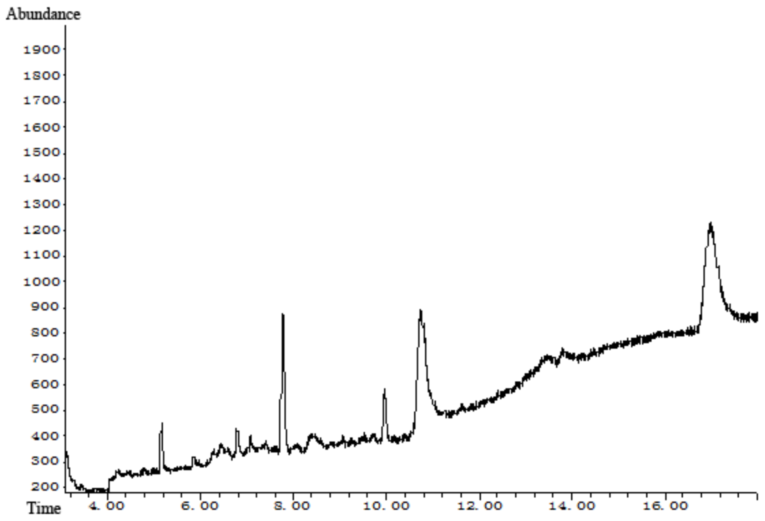

(B)

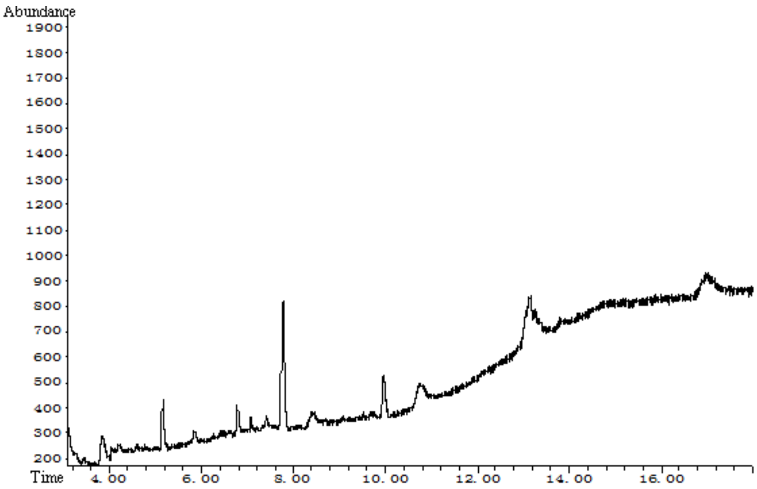
Abundance
1900
1800
1700
1600
1500
1400
1300
1200
1100
1000
900
800
700
600
500
400
300
200
Time
4.00
6.00
8.00
10.00
12.00
14.00
16.00

(C)

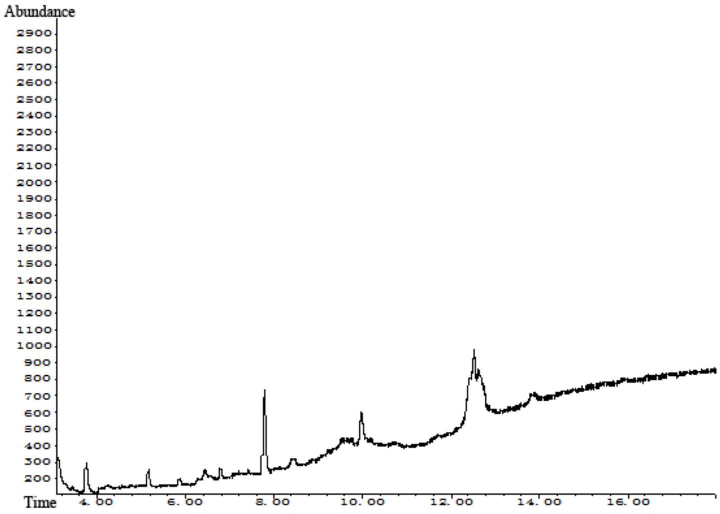
Abundance
2900
2800
2700
2600
2500
2400
2300
2200
2100
2000
1900
1800
1700
1600
1500
1400
1300
1200
1100
1000
900
800
700
600
500
400
300
200
Time
4.00
6.00
8.00
10.00
12.00
14.00
16.00

(D)
